# NRP-1 Receptor Expression Mismatch in Skin of Subjects with Experimental and Diabetic Small Fiber Neuropathy

**DOI:** 10.1371/journal.pone.0161441

**Published:** 2016-09-06

**Authors:** Nathalie Van Acker, Michael Ragé, Hilde Vermeirsch, Dorien Schrijvers, Rony Nuydens, Geert Byttebier, Maarten Timmers, Stefanie De Schepper, Johannes Streffer, Luc Andries, Léon Plaghki, Patrick Cras, Theo Meert

**Affiliations:** 1 Faculty of Medicine and Health Sciences, University of Antwerp, Antwerp, Belgium; 2 HistoGeneX NV, Antwerp, Belgium; 3 Institute of Neuroscience, Université Catholique de Louvain, Brussels, Belgium; 4 Janssen Research and Development, Janssen Pharmaceutics NV, Beerse, Belgium; 5 Reference Center for Biological Markers of Dementia (BIODEM), Institute Born-Bunge, University of Antwerp, Antwerp, Belgium; 6 Department of Neurology, Antwerp University Hospital, Born Bunge Institute, University of Antwerp, Antwerp, Belgium; Hirosaki Daigaku, JAPAN

## Abstract

The *in vivo* cutaneous nerve regeneration model using capsaicin is applied extensively to study the regenerative mechanisms and therapeutic efficacy of disease modifying molecules for small fiber neuropathy (SFN). Since mismatches between functional and morphological nerve fiber recovery are described for this model, we aimed at determining the capability of the capsaicin model to truly mimic the morphological manifestations of SFN in diabetes. As nerve and blood vessel growth and regenerative capacities are defective in diabetes, we focused on studying the key regulator of these processes, the neuropilin-1 (NRP-1)/semaphorin pathway. This led us to the evaluation of NRP-1 receptor expression in epidermis and dermis of subjects presenting experimentally induced small fiber neuropathy, diabetic polyneuropathy and of diabetic subjects without clinical signs of small fiber neuropathy. The NRP-1 receptor was co-stained with CD31 vessel-marker using immunofluorescence and analyzed with Definiens® technology. This study indicates that capsaicin application results in significant loss of epidermal NRP-1 receptor expression, whereas diabetic subjects presenting small fiber neuropathy show full epidermal NRP-1 expression in contrast to the basal expression pattern seen in healthy controls. Capsaicin induced a decrease in dermal non-vascular NRP-1 receptor expression which did not appear in diabetic polyneuropathy. We can conclude that the capsaicin model does not mimic diabetic neuropathy related changes for cutaneous NRP-1 receptor expression. In addition, our data suggest that NRP-1 might play an important role in epidermal nerve fiber loss and/or defective regeneration and that NRP-1 receptor could change the epidermal environment to a nerve fiber repellant bed possibly through Sem3A in diabetes.

## Introduction

The *in vivo* cutaneous nerve regeneration model, using capsaicin, the most abundant pungent molecule produced by pepper plants [1], has been used extensively to study the mechanisms underlying neuropathic pain syndromes. Capsaicin is widely used in the treatment of neuropathic and musculoskeletal pain [2–4] and for studying nerve regeneration therapies [5]. The capsaicin model, as applied in the previously published paper [6], was developed and standardized by Polydefkis et al. [7]. When applied topically to the skin, a reversible superficial skin denervation occurs which is referred to as chemical axotomy. The neurotoxic properties of capsaicin exist through its activation of the transient receptor potential vanilloid type 1 (TRPV1) present on the free small nerve fiber endings in skin. Chronic activation of this receptor results in desensitization and degeneration of the epidermal nerve fibers [2,8]. After discontinuation of the neurotoxic application however, the fibers regenerate rapidly, resulting in a dynamic mimicking of the development and reversal of small fiber neuropathy (SFN) [9–11]. Morphologically, the rapid and profound loss of the epidermal nerve fibers is proven in these studies using the pan-axonal marker PGP9.5, the ‘gold standard’ to visualize the number and morphology of the somatic, small caliber, unmyelinated intra-epidermal nerve fibers as supported by the European Federation of Neurological Science [12]. In previously published work however, we described a mismatch between functional and morphological nerve fiber recovery in the capsaicin model [6]. Therefore, the question arose whether this model represents the clinical status of mechanisms involved in SFN, e.g. in diabetes [13–21]. Candidate biomarkers are defined to evaluate the model’s suitability to study SFN’s progression and cure.

Diabetes mellitus (DM) is the most common identifiable cause of SFN; however, sometimes, SFN may be idiopathic in nature [22]. It is believed that the pathophysiological mechanisms of diabetic neuropathy are related to hyperglycemia [23], polyol pathway hyperactivity, microvascular changes, sodium-channel hyper activation, oxidative and nitrosative stress [24], accumulation of glycation end products [25], impaired calcium-homeostasis [26] and mitochondrial dysfunction [27,28]. As microangiopathy is related to diabetic neuropathy, it is also known to lead to foot ulceration and amputation of the lower limbs [29,30]. Though the precise mechanism of this process is poorly understood, it is proposed that interplay among endothelial dysfunction, impaired nerve axon reflex activities, and microvascular regulation due to hyperglycemia in diabetes may play a significant role [31]. Hyperglycemia leads to reduced supply of oxygen to nerves and tissues; hypoxia impedes the viability of tissues and the wound healing process [32,33] and induces the expression of several angiogenic genes, most notably vascular endothelial growth factor (VEGF) through the HIF-1 pathway [34–36]. The HIF-1-mediated transactivation and subsequent angiogenic abilities are functionally inhibited in diabetes, resulting in an impairment to restore blood flow to ischemic regions [35,37,38] and leading to vascular complications [39]. The microvascular complications in diabetes have been shown to be related to C-fiber dysfunction as well [40]. Nerve and blood vessel growth and regenerative capacities are regulated through the neuropilin-1 (NRP-1) and semaphorin pathway [41,42]. The NRP-1 receptor, originally discovered as a receptor for the axon guidance factors belonging to the class-3 semaphorin subfamily, enhances VEGF signaling during vasculogenesis and angiogenesis [43]. Loss of endothelial NRP-1 results in vessel remodeling and branching defects [44–46]. Many molecular signals, including NRP-1, are shared by nerve and blood vessel growth for purposes of navigation, regeneration and arborization [47] indicating an intriguing resemblance between nerve and blood vessel networking [48,49]. Mukouyama and colleagues described that peripheral nerve-induced arteriogenesis exists through the VEGF-NRP-1 positive feedback-loop [50]. In the present study we evaluated the ability of the capsaicin model to mimic the cutaneous microenvironment of patients with diabetic polyneuropathy by studying NRP-1 receptor expression in epidermis, dermal vasculature and dermal non-endothelial structures.

## Materials and Methods

### Human samples

Studies conducted by Ragé and colleagues from which biopsies were used; were approved by the Local Ethics Committee of the ‘Université Catholique de Louvain’. References and inclusion criteria can be obtained from previously published work [6,51]. Written informed consent was obtained from all participants. In the experimental capsaicin model, 9 healthy volunteers received a topical capsaicin application on three consecutive 24-hour cycles (total 72 hours). This was performed according to the methods used by Polydefkis [7] as previously described [6]. Skin biopsies were assessed at day 1 (D1, n = 9), at a variable time point that was either on day 12 or day 26 (DVar, n = 9) and at day 54 (D54, n = 9) after capsaicin was applied. Subjects underwent a screening evaluation to assess their eligibility for study participation. The screening evaluation included a medical history, concomitant medication recording, physical and neurological examination and routine laboratory testing. Within 14 days prior to the start of capsaicin application, baseline Quantitative Sensory Testing (QST), CO_2_ laser stimulation and skin punch biopsies were performed to serve as a control (n = 9). Successful degeneration and regeneration of epidermal nerve fibers in this capsaicin model were confirmed in our former study by means of standard PGP9.5 immunofluorescence staining and IENF density assessment [6]. Additionally, biopsy specimens from healthy subjects (n = 3), diabetic subjects with poly-neuropathy (diabetes mellitus; DM Type 2 NP+, n = 5) and diabetic subjects without conventional clinical signs of neuropathy (DM Type 1, n = 8; DM Type 2, n = 8) were obtained from an earlier study. Two outpatient visits to the clinical unit were planned on a time-span of maximum 2 weeks, with a minimum interval of 3 days. Patients underwent a screening evaluation including medical history, concomitant medication recording, physical and neurological examination. The Toronto Clinical Scoring System (TCSS) was used to screen for diabetic neuropathy. None of the control subjects had a history of alcohol or drug abuse, significant illnesses, or clinical findings suggestive of peripheral or central nervous system disorders [51].

### Skin punch biopsy assessment and processing

During the studies conducted by Ragé and colleagues [6,51] skin biopsies were processed according to the standard skin biopsy procedure for IENF density assessment and in parallel according to the method described here. Skin punch biopsies (3–4 mm in diameter; Biopsy punch, Kai Europe GmbH, Germany) were performed under local anesthesia at the lateral aspect of the distal leg in a clinical unit. Skin biopsies were snap-frozen in liquid nitrogen after protecting with optimum cutting tissue (OCT) compound (Sakura TissueTek Europe, The Netherlands). After freezing, tissue-blocks were sectioned at 2 levels and 12 μm thickness. Cryosections were collected on dry ice and subsequently fixed in formalin-based fixative, prior to staining. This additional skin biopsy processing method allowed us to evaluate the expression of other targets than PGP9.5, otherwise incompatible with the standard skin biopsy procedure.

### NRP-1 antibody accuracy by western blot analysis and immunohistochemistry

Western blot analysis was performed by SDS-page on HEK293 cell line lysates exhibiting no or high NRP-1 expression (kindly provided by Roche, Penzberg), to verify the accuracy of the goat polyclonal anti-human NRP-1 antibody (Clone C-19, Santa Cruz Biotechnology, Inc., USA; 1/200). Subsequently, western transfer of proteins was performed onto a transfer membrane (Immobilon FL, Li-Cor Biosciences, USA). After incubation of the anti-human NRP-1 antibody, visualization was performed using IRDye® 800CW Conjugated Rabbit anti-Goat IgG antibody on the Odyssey® Infrared Imaging System (all Li-Cor Biosciences).

The Labvision autostainer (LabVision, Thermo Fisher Scientific, USA) was used for NRP-1 immunohistochemical staining of formalin fixed paraffin embedded cell preparations. Wild type and NRP-2 transfected HEK293 cells, which should exhibit baseline NRP-1 expression; and NRP-1 transfected HEK293 cells, which should exhibit high NRP-1 expression, are included (Roche, Penzberg). After heat induced epitope retrieval using Citrate buffer (pH 6.0, LabVision), slides were incubated with the primary goat anti-human NRP-1 antibody (Clone C-19, Santa Cruz Biotechnology, Inc., USA; 1/700). Afterwards, a secondary antibody (Rabbit anti-Goat IgG, Invitrogen, Thermo Fisher Scientific, USA) was applied to recognize the primary antibody, and subsequently, a labeled polymer (EnVision+ System- HRP Labeled Polymer Anti-Rabbit, Agilent Technologies, Dako, Denmark) was used to recognize the secondary antibody. DAB+ (Liquid DAB, Agilent Technologies) was used for visualization. As a negative control, normal goat immunoglobulins (Santa-Cruz Biotechnology, Inc., USA; 1/1400) were used to replace the primary antibody. Nuclear counterstain was performed using Hematoxylin (Agilent Technologies).

### CD31 and NRP-1 double labeling immunofluorescence

Skin tissue sections were incubated overnight at room temperature using a mix of the mouse monoclonal anti-human CD31 primary antibody (clone JC70A, Agilent Technologies; 1/250) and the goat polyclonal anti-human Neuropilin-1 receptor antibody (Clone C-19, Santa Cruz Biotechnology; 1/25). Subsequently, visualization of the target antibodies was established using a Cy3-labeled secondary donkey anti-goat antibody and thereafter an Alexa-488 labeled goat anti-mouse antibody (both obtained from Jackson Immunoresearch Laboratories, USA; 1/100 and 1/500 respectively) incubation. To enhance penetration of immunoreagents into the sections, tissue was permeabilized using TritonX100 (Sigma-Aldrich, USA; 0.1%). As a negative control, host immunoglobulins (Confirm Negative control mouse, Ventana, Roche, USA; Goat immunoglobuline G, R&D Systems, USA) were used to replace the primary antibody. Counterstain was performed using Hoechst 33342 (Invitrogen Molecular Probes, Thermo Fisher Scientific, USA; 1/2000).

### Imaging and Quantification

Virtual images were generated using the Axiovision Mozaik Imaging Software (Axiovision Rel 4.8) on an Axioplan 2 Imaging microscope equipped with motorized stage and z-stack features (Carl Zeiss, Germany) using the same exposure times for all channels and samples. Morphological evaluation was performed on the virtual images with descriptive outcome. Image analysis and quantification of NRP-1 receptor expression in skin biopsies was developed and performed using the software package Definiens® Developer and Architect XD^TM^ 64 (Definiens®, Germany) for both epidermis and dermis.

**Fig 1:**
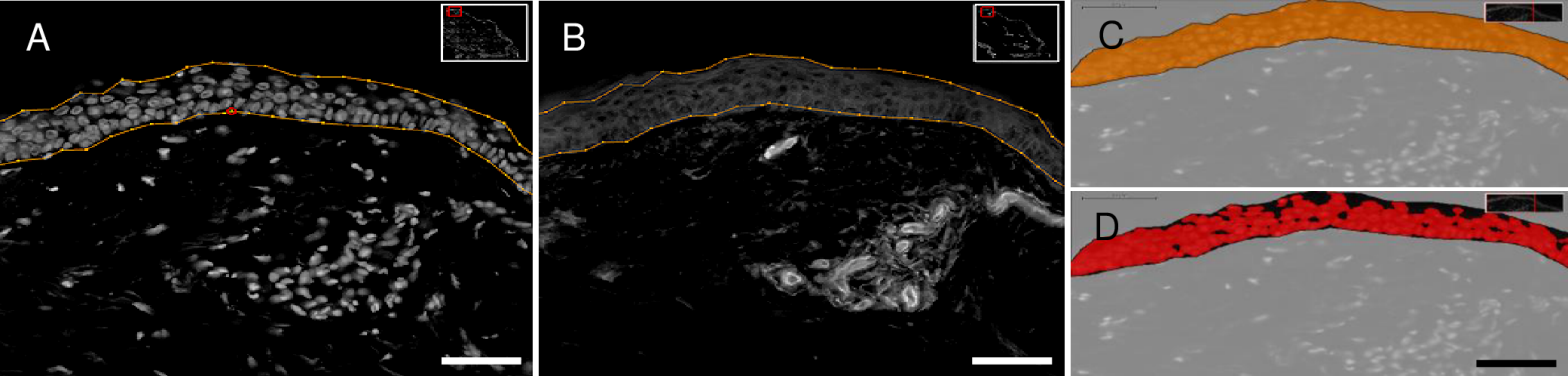
Definiens® analysis of NRP-1 expression in epidermis. (A) Selection of epidermal area using Hoechst nuclear counterstain. (B) Selected epidermal area shown in NRP-1 layer. (C) Total epidermal area in orange. (D) NRP-1 positive marker area as recognized by software in red. Scale bar: 50μm.

To study the epidermis, a NRP-1 marker area analysis was performed relative to the total epidermal region and expressed as percentage. The epidermis was manually delineated following the dermal-epidermal junction and covering the nucleated epidermal layers (Fig 1A). The analysis method considered signals being positive when signals above background were present. Background level was determined using the IgG isotype control. Each image is built of two individual layers, one for each fluorescent tag (Fig 1A: Layer 1 for Hoechst; Fig 1B: Layer 2 for NRP-1), used for marker area analysis. A specific rule set was written in Definiens® Developer XD^TM^ 64 to determine the % marker area positive for NRP-1 (Fig 1D) in epidermis (Fig 1C).

**Fig 2:**
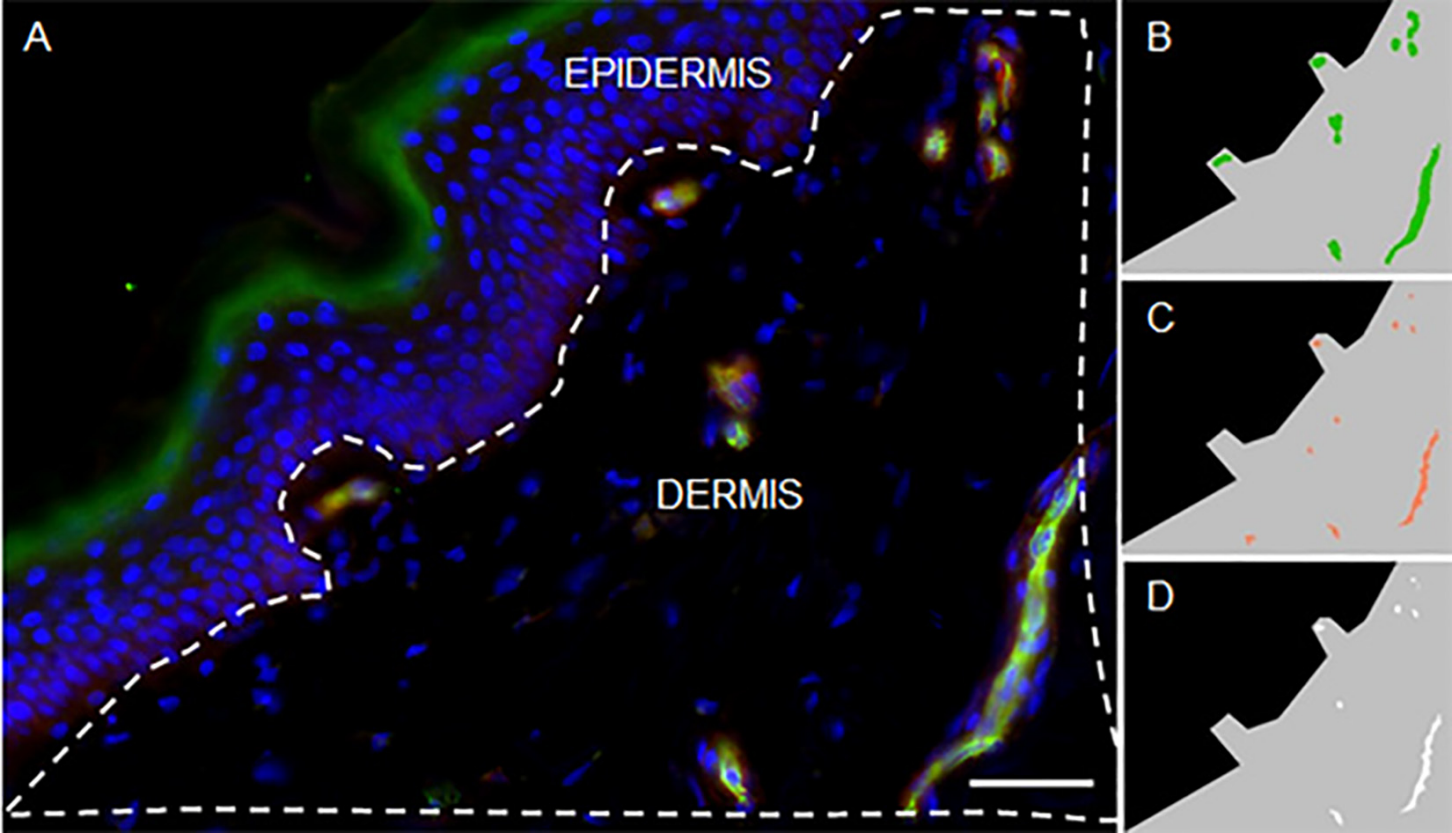
Definiens® analysis of NRP-1 expression in dermis. (A) NRP-1 (red)/CD31 (green) double labeling immunofluorescence in the dermis of human skin biopsy. Dashed line represents the delineation of the ROI in dermis. (B) NRP-1 (green) immunoreactivity subtracted by Definiens® technology in delineated ROI (grey). (C) CD31 (orange) immunoreactivity subtracted by Definiens® technology in delineated ROI (grey). (D) NRP-1/CD31 double staining (white) subtracted by Definiens® technology in delineated ROI (grey). Scalebar: 50μm.

Image analysis and quantification of dermal number and area of NRP-1, CD31 and NRP-1/CD31 double stained structures was performed using the software package Definiens® Developer and Architect XD^TM^ 64 (Definiens®, Germany).This allowed automated analysis of a large region of interest (ROI), in each tissue section (Fig 2A). The ROI in the dermis (Fig 2B–2D, grey area) was manually delineated at the dermal-epidermal junction up to 300 μm towards the hypodermis. The analysis method considered signals being positive when signals above background were present. Background level was determined using the IgG isotype control. Each image is built of three individual layers, one for each fluorescent tag (Layer 1: Hoechst; Layer 2: CD31; Layer 3: NRP-1), used for segmentation and classification during the automated analysis. All stained structures within the ROI were counted and expressed as number per mm^2^. The stained area relative to the total ROI analyzed was determined and expressed as percentage area/ROI. Accessory structures and staining artifacts were excluded from each ROI. To study vascular NRP-1 expression in the dermis, a co-localization experiment was performed using the universal vascular marker CD31. A specific rule-set was written in Definiens® Developer XD^TM^ 64 to determine NRP-1^+^ (Fig 2B, green), CD31^+^ (Fig 2C, orange), and combined (CD31^+^/NRP-1^+^ co-localization^+^, Fig 2D, white) immunofluorescence staining. The dimension and size of the CD31-immunoreactive vessels was determined using the form-factors ‘sum of length’ and ‘sum of border’ of these vessels, relative to the area.

### Statistics

Statistical analysis was performed using MedCalc® v11.2.1 statistical software. An ANOVA Friedman, non-parametrical test was used for analysis of data obtained from the capsaicin model. All other data were analyzed using Kruskal-Wallis test. P-values < 0.05 were considered significant.

## Results

### Demographic data

Selection of participants can be consulted in published studies conducted by Ragé and colleagues [6,51]. Findings of these studies are summarized here for completeness.

All subjects enrolled for the experimental SFN study had normal QST and LEPs at the screening evaluation and were invited to receive a topical capsaicin application. At day 1 post-capsaicin, cutaneous thermal sensitivity was reduced, as were LEPs; PGP9.5, TRPV1 and GAP-43 immunoreactive nerve fibers were almost completely absent confirming successful degeneration of epidermal nerve fibers [6].

For diabetics, patients were divided into three groups (Table 1): DM Type 1 patients, DM Type 2 patients; both without clinical manifestations of neuropathy; and DM Type 2 patients with neuropathy (DM Type 2 NP+). Mean age was significantly different between groups as a direct consequence of the inclusion criteria (p <0.05) with DM Type 1 patients being significantly younger as described in our previous manuscript [51]. The groups did not differ in height and mean BMI was significantly higher in DM Type 2 (p <0.05). Known diabetes duration, current glycated hemoglobin level (HbA1C) and serum creatinine was similar between both subtypes of diabetic patients, including patients with overt diabetic neuropathy (DNP). As per protocol, none of both subtypes of DM patients Type 1 and 2 had neuropathic symptoms. All these patients scored < 4 on the Toronto Clinical Scoring System for Diabetic Polyneuropathy, indicating that there was no clinical evidence for DNP. Conversely, all patients with DNP had per protocol a TCSS > 5 and < 10 (mild to moderate DNP) [51].

**Table 1:** Baseline demographics.

	Controls	DM Type 1	DM Type 2	DM Type 2 NP+
Sex (F/M)	3/9	2/6	3/5	0/5
age, mean [SD]	44.50 [8.97]	33.75 [3.20] *	56.33 [3.54] *	55.4 [3.29] *
height(cm), mean [SD]	175.00 [10.27]	176.63 [8.63]	168.33 [10.92]	172.00 [6.78]
BMI, mean [SD]	26.35 [4.30]	25.00 [4.10]	30.54 [2.26] *	28.20 [2.60]
DM duration (years), mean [SD]	NA	18.90 [8.76]	13.78 [3.50]	16.20 [4.60]
Daily cumulative insulin, mean [SD]	NA	53.5 [9.27]	60.56 [64.22]	37.00 [25.53]
IDF score, mean [SD]	NA	0.63 [0.92] *	4.00 [1.32]	3.60 [0.55]
Creatinin, mean [SD]	NA	0.85 [0.10]	0.92 [0.15]	0.78 [0.17]
HbA1C, mean [SD]	NA	8.25 [1.13]	8.14 [1.42]	9.24 [0.94]
Toronto score, mean [SD]	NA	0.38 [0.74]	0.78 [1.09]	7.20 [2.28]
IENFd, mean [SD]	13.80 [7.02]	8.70 [5.40]	6.40 [3.30]	1.90 [3.40]

BMI = body mass index; DM = Diabetes mellitus; IDF = International Diabetes Federation score; HbA1C = glycated hemoglobin; NA = not applicable; NP = neuropathy; IENFd = Intra-epidermal nerve fiber density; SD = Standard Deviation.

*p < 0.05

### Accuracy of the NRP-1 antibody

**Fig 3:**
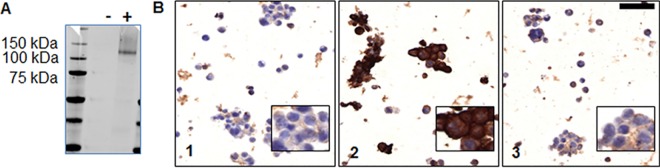
Accuracy of NRP-1 receptor antibody. (A) Western blot analysis of NRP-1 transfected (+) HEK293 cells and wild-type (-) HEK293 cells. (B) NRP-1 immunohistochemistry performed on wild type (B1), NRP-1- (B2) and NRP-2- (B3) transfected HEK293 cells. Scale bar: 50μm.

Western blot analysis of cell lysates of NRP-1 transfected HEK293 cells showed a specific band for NRP-1 at the expected molecular weight of approximately 130 kDa. For the wild-type HEK293 cell control no such band is present (Fig 3A). Specificity of the antibody used in this study was further strengthened using immunohistochemical staining of wild type, NRP-1- and NRP-2 transfected HEK293 cells. Wild type and NRP-2 transfected HEK293 cells showed basal (weak) staining for NRP-1 in the applied settings, whereas NRP-1 transfected HEK293 cells showed a very intense cytoplasmic labeling of the protein (Fig 3B). The negative control for which goat immunoglobulins were used lacked immunoreactivity (data not shown).

### NRP-1 receptor expression in skin of healthy controls

**Fig 4:**
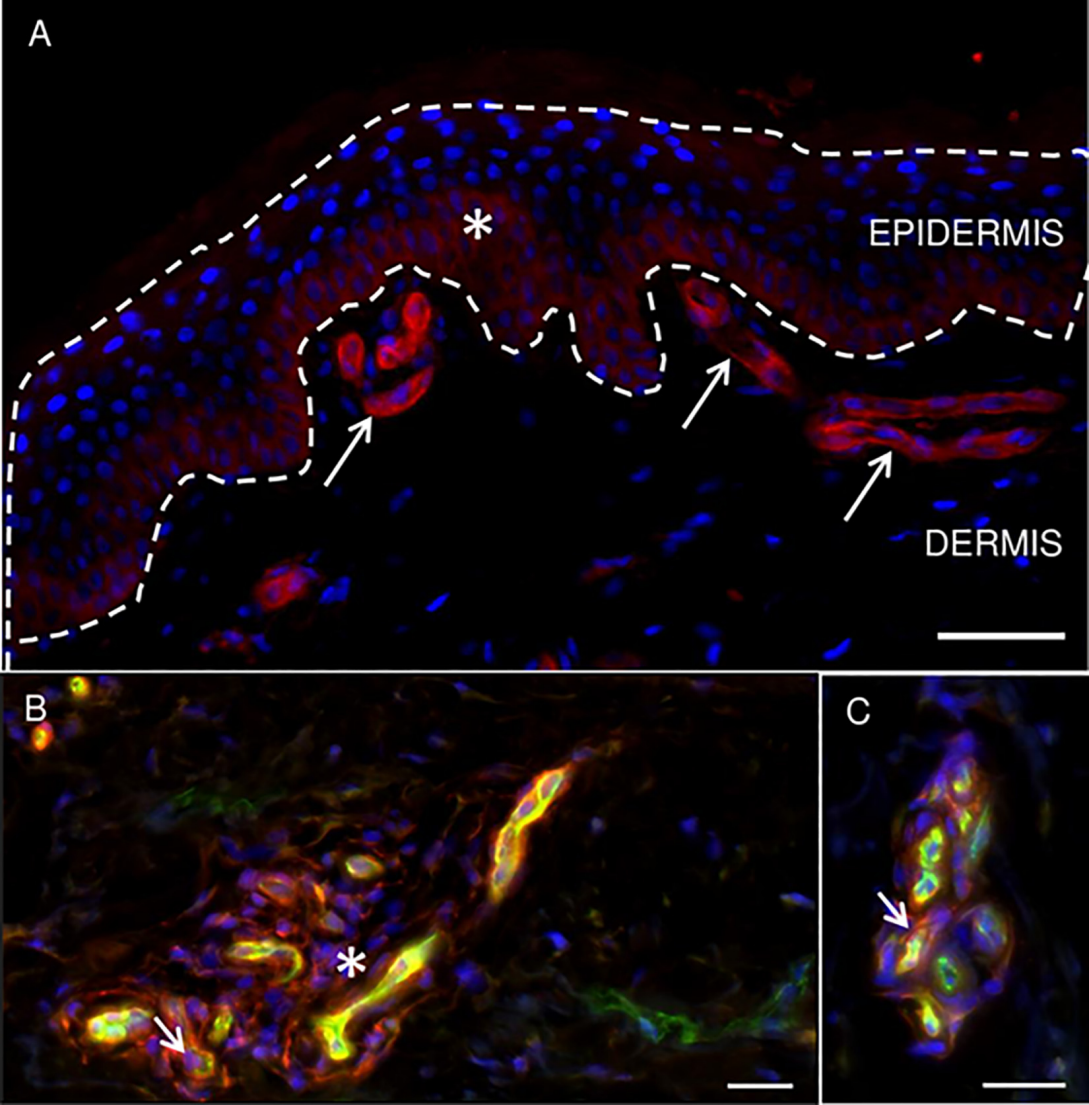
NRP-1 receptor expression related to epidermis and dermal vasculature in skin of healthy volunteers. (A) NRP-1 receptor expression in human skin biopsy. NRP-1 was present in dermal vasculature (arrows) and basal keratinocytes of epidermis (asterix). (B and C) NRP-1 receptor (red) and CD31 (green) expression patterns in dermis of human skin biopsy. NRP-1 was present in dermal vasculature (yellow, co-localization of NRP-1 and CD31), adjacent to vessels (arrow; CD31, green) and in cells with a fibroblastic phenotype (asterix). Hoechst counterstain (blue). Scalebar: 20μm.

As shown in Fig 4A, the NRP-1 receptor was found to be expressed in the epidermal basal layers in skin of all healthy controls. Additional weak NRP-1 immunoreactivity was observed in the supra-basal layers of the epidermis in 25% of healthy subjects (3/12). A prominent but variable NRP-1 expression was found in dermal vasculature and non-endothelial cells adjacent to vessels. Cells presenting a fibroblastic phenotype present in the dermis showed NRP-1 expression as well (Fig 4B and 4C). No specific markers were used however to identify the cell types exhibiting NRP-1 expression.

### Capsaicin application results in a loss of epidermal NRP-1 receptor expression, whereas a full epidermal NRP-1 receptor expression is present in diabetic subjects with neuropathy

**Fig 5:**
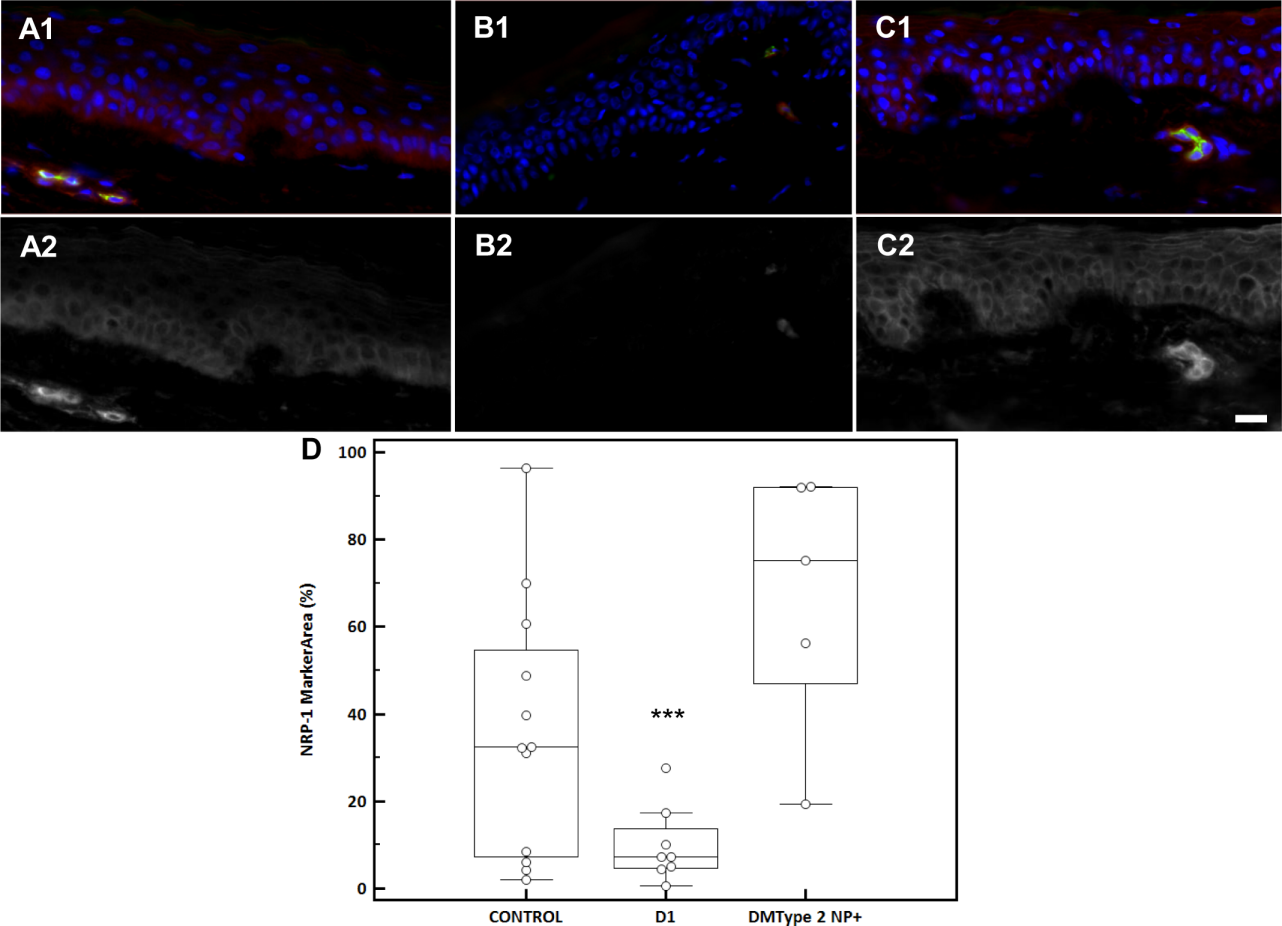
NRP-1 Receptor expression in the epidermis of control, D1 capsaicin treated and DM Type 2 NP+ subjects. NRP-1 receptor (red) and CD31 (green) expression pattern in epidermis as obtained by NRP-1/CD31 immunofluorescence in human skin biopsy of (A) healthy subject,(B) capsaicin treated healthy subject on day 1 (D1), (C) DM Type 2 NP+ diabetic subject. The lower panel (A2, B2, and C2) shows a grey scale image of NRP-1. Hoechst nuclear counterstain (blue). Scalebar: 20μm. (D) Multiple comparison graph of NRP-1 Marker area (%) in epidermis of control, D1 and DM Type 2 NP+ subjects. * p < 0.05.

Morphological analysis of skin biopsies from healthy volunteers who received topical capsaicin (D1) revealed that 78% (7/9) did not show epidermal NRP-1 receptor expression in contrast to controls (Fig 5A and 5B). On day 54 after capsaicin was applied (D54); NRP-1 receptor expression appeared again in basal epidermal layers in 67% (6/9) of cases. However, in epidermis of diabetic subjects suffering from DNP, a prominent NRP-1 expression was present on basal and supra-basal epidermal cells (Fig 5C) showing a membranous staining pattern (90%, 4/5). These results indicate that capsaicin has a negative impact on epidermal NRP-1 expression, whereas the receptor appears in the entire epidermis in diabetic subjects presenting DNP. The automated Definiens® marker area analysis confirmed a significant reduction of NRP-1 receptor expression in capsaicin-treated skin (p = 0.009; 10% [10%]) compared to controls (36% [17%]) and diabetic subjects presenting DNP (67% [11%]; Fig 5D). An increase of the NRP-1 epidermal marker area was seen in diabetic subjects compared to controls which was prominent but not significant (p = 0.092).

### Dermal NRP-1-expression is decreased in capsaicin-induced SFN but not in diabetes

**Fig 6:**
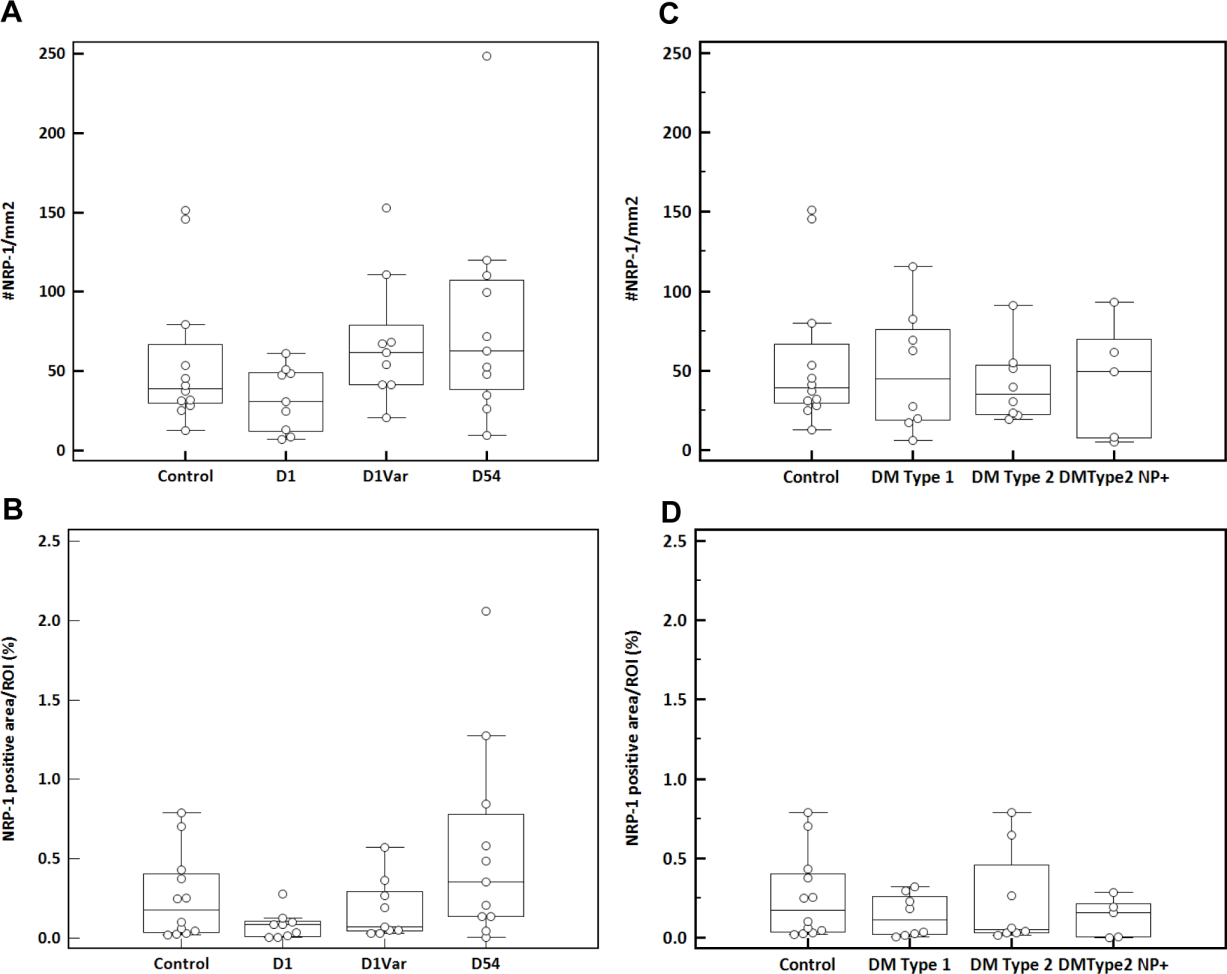
Definiens analysis of NRP-1 receptor expression in dermis. (A) Number of NRP-1 positive structures (#/mm^2^) and (B) NRP-1 positive area/ROI (%) in dermis of controls, healthy volunteers who received topical capsaicin at day 1 (D1), variable time point (Dvar) and day 54 (D54) after capsaicin was applied. (C) Number of NRP-1 positive structures (#/mm^2^) and (D) NRP-1 positive area/ROI (%) in dermis of controls, diabetic subjects Type 1 (DM Type 1), Type 2 (DM Type 2) and diabetic subjects suffering from polyneuropathy (DM Type 2 NP+).

Skin biopsies of healthy volunteers who received topical capsaicin showed a marked decrease in the mean density of dermal NRP-1 stained structures at D1 (32.6+/-20.3/mm^2^) compared to controls (p = 0.08; 52.3+/-45.9/mm^2^). The density of dermal NRP-1 stained structures recovered and increased from DVar (75.4+/-40.1/mm^2^) to D54 (90.2+/-68.1/mm^2^; Fig 6A; Table 2). Accordingly, a marked decrease was observed in the percentage of the dermal stained area for NRP-1 at D1 (0.08+/-0.09%) compared to controls (0.29+/-0.27%), recovering by DVar (0.30+/-0.19%). This was reflected by a significant increase (p = 0.04) in the percentage of the stained area for NRP-1 between D1 (0.08+/-0.09%) and D54 (0.54+/-0.63%) (Fig 6B, Table 2). No significant difference was seen in the dermal NRP-1 positive area in skin of diabetic subjects as compared to controls (p = 0.56; Control: 0.29+/-0.27%; DM Type 1: 0.14+/-0.13%; DM Type 2: 0.23+/-0.31%; DM Type 2 NP+: 0.13+/-0.12%; Fig 6D). Concomitantly, no difference was detected in the density of NRP-1 labeled structures (p = 0.91) in any group (Control: 52.3+/-45.9/mm^2^; DM Type 1: 50.1+/-38.5/mm^2^; DM Type 2: 41.6+/-24.2/mm^2^; DM Type 2 NP+: 43.5+/-37.3/mm^2^) (Fig 6C; Table 2). Evidence is provided that capsaicin application to the skin results in a decrease of the NRP-1 receptor in dermis of healthy subjects to recover by D54 after capsaicin was applied. Statistical significance was only reached between D1 and D54. None of the diabetic subjects showed changes in the dermal NRP-1 expression. These data indicate that the dermal microenvironment in the capsaicin model does not resemble that of diabetic neuropathy, with regards to dermal NRP-1 expression.

**Table 2:** Definiens analysis results for dermal NRP-1 Receptor expression.

	Variables	Control	D01	DVar	D54	DM Type 1	DM Type 2	DM Type 2 NP+
n		12	9	9	9	8	8	5
CD31	number/ROI	117.6	88.8	103.9	132.5	114.7	104.1	111.6
	[SD]	[72.2]	[30.0]	[33.1]	[64.8]	[43.5]	[20.0]	[49.1]
	length/ROI	0.0008	0.0006	0.0007	0.001	0.0007	0.0008	0.0009
	[SD]	[0.0005]	[0.0002]	[0.0002]	[0.0005]	[0.0002]	[0.0003]	[0.0005]
	border/ROI	0.003	0.002	0.003	0.004	0.003	0.003	0.004
	[SD]	[0.002]	[0.001]	[0.0008]	[0.002]	[0.0008]	[0.0009]	[0.002]
	area/ROI(%)	0.17	0.13	0.15	0.21	0.14	0.20	0.25
	[SD]	[0.11]	[0.08]	[0.04]	[0.15]	[0.04]	[0.08]	[0.15]
NRP1	number/ROI	52.3	32.6	75.4	90.2	50.1	41.6	43.5
	[SD]	[45.9]	[20.3]	[40.1]	[68.1]	[38.5]	[24.2]	[37.3]
	area/RO I(%)	0.29	0.08*	0.30	0.54*	0.14	0.23	0.13
	[SD]	[0.27]	[0.09]	[0.19]	[0.63]	[0.13]	[0.31]	[0.12]
NRP1/CD31	number/ROI	104.4	88.9	89.3	118.5	68.4	110.3	100.3
	[SD]	[54.1]	[46.8]	[26.1]	[54.4]	[36.0]	[42.4]	[84.7]
	area/ROI(%)	0.05	0.03	0.04	0.06	0.03	0.06	0.07
	[SD]	[0.04]	[0.02]	[0.02]	[0.05]	[0.02]	[0.03]	[0.05]

CD = Cluster of differentiation; DM = diabetes mellitus; DX = day X after capsaicin is applied; NP+ = presenting polyneuropathy; NRP-1 = Neuropilin-1 receptor; ROI = region of interest; SD = Standard Deviation; Var = variable

* indicates significance.

### Vascular NRP-1 receptor expression remained unchanged in both capsaicin-induced SFN and diabetes

**Fig 7:**
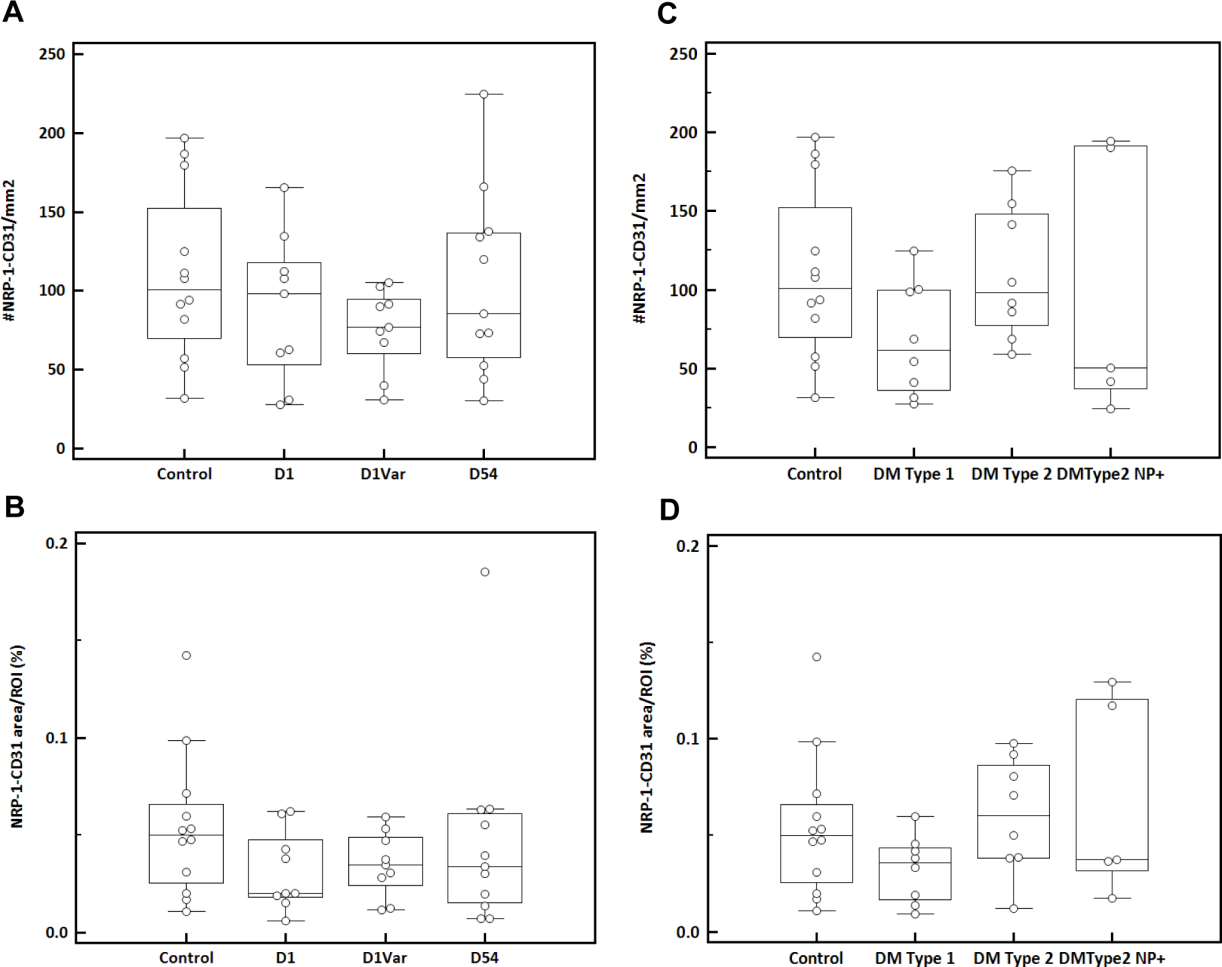
Definiens analysis of vascular NRP-1 receptor expression in dermis. (A) Number of NRP-1/CD31 positive structures (#/mm^2^) and (B) NRP-1/CD31 positive area/ROI (%) in dermis of controls, healthy volunteers who received topical capsaicin at day 1 (D1), a variable time point (Dvar) and day 54 (D54) after capsaicin was applied. (C) Number of NRP-1/CD31 positive structures (#/mm^2^) and (D) NRP-1/CD31 positive area/ROI (%) in dermis of controls, diabetic subjects Type 1 (DM Type 1), Type 2 (DM Type 2) and diabetic subjects suffering from polyneuropathy (DM Type 2 NP+).

No significant difference was seen for dermal NRP-1/CD31 double positive-vessel density during the time course after capsaicin was applied (p = 0.17; control: 104.4+/-54.1/mm^2^; D1: 88.9+/-46.8/mm^2^; DVar: 89.3+/-26.1/mm^2^; 118.5+/-54.4/mm^2^; Table 2). The NRP-1/CD31 double positive stained area remained unchanged as well (p = 0.37; control: 0.05+/-0.04%; D1: 0.03+/-0.02%; DVar: 0.04+/-0.02%; D54: 0.06+/-0.05%; Fig 7A and 7B). Similarly, in diabetic subjects, the density of NRP-1 positive vessels was not different from the density seen in healthy controls (p = 0.30; 104.4+/-54.1/mm^2^), for those who are asymptomatic for neuropathy (DM Type 1 (68.4+/-36.0/mm^2^; DM Type 2 (110.3+/-42.4/mm^2^) and those with diabetic polyneuropathy (DM Type 2 NP+: 100.3+/-84.7/mm^2^) (Fig 7C). This reflected in a comparable NRP-1/CD31 double positive stained area measured in these biopsies (p = 0.32; control: 0.05+/-0.04%; DM Type 1: 0.03+/-0.02%; DM Type 2: 0.06+/-0.03%; DM Type 2 NP+: 0.07+/-0.05%; Fig 7D). These results indicate that vascular NRP-1 receptor expression remained unchanged in capsaicin treated skin and skin of diabetic subjects presenting DNP. The capsaicin-induced changes in dermal NRP-1 expression are therefore related to non-endothelial cells.

### Repeated capsaicin application induces vasoconstriction, whereas no changes were observed in dermal vasculature of diabetic subjects

**Fig 8:**
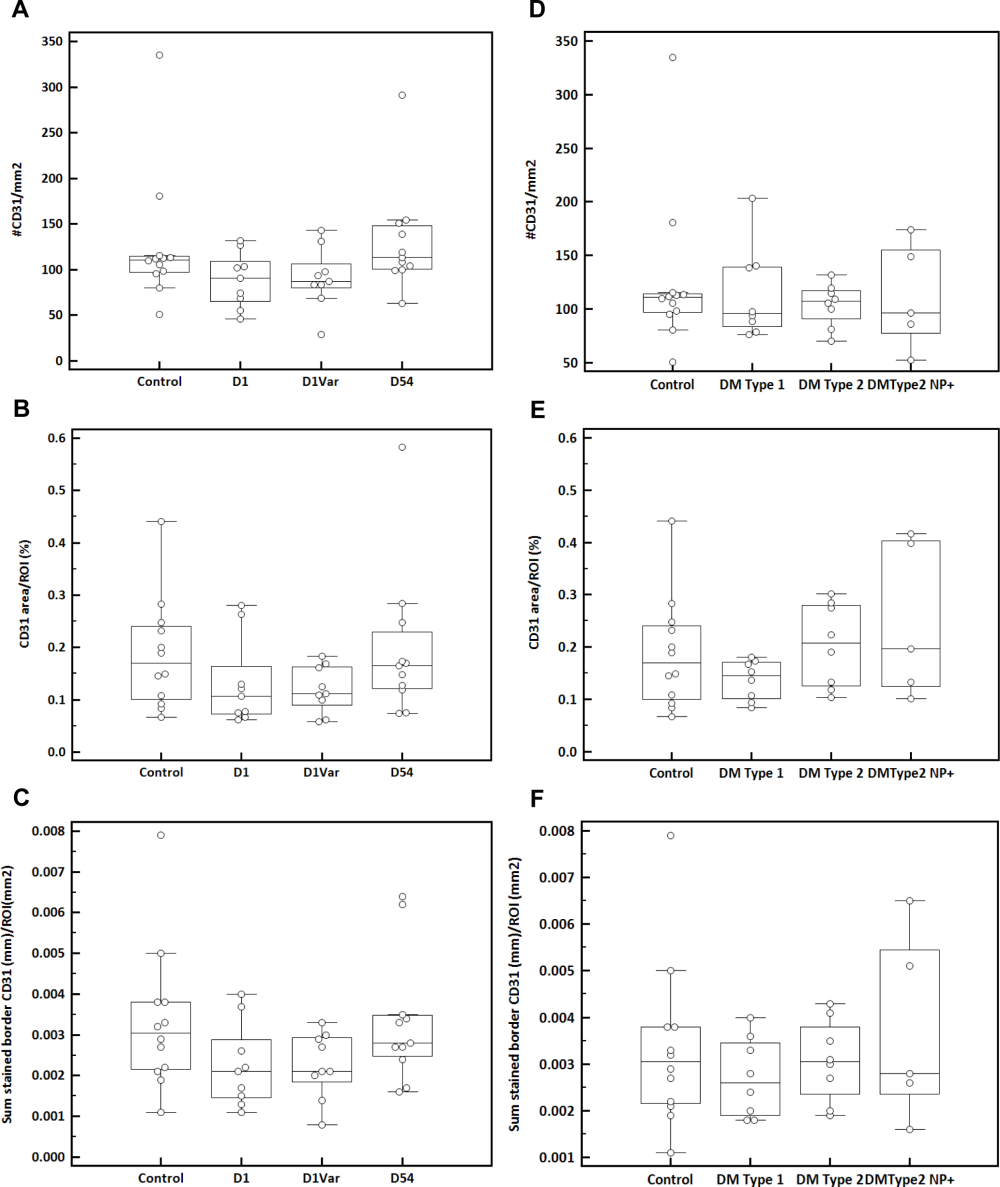
Definiens analysis of dermal vasculature. (A) Number of CD31 positive structures (#/mm2); (B) CD31 positive area/ROI (%); (C) Sum of CD31 positive border in dermis of controls, healthy volunteers who received topical capsaicin at day 1 (D1), a variable time point (Dvar) and day 54 (D54) after capsaicin was applied. (D) Number of CD31 positive structures (#/mm2); (E) CD31 positive area/ROI (%); (F) Sum of CD31 positive border in dermis of controls, diabetic subjects Type 1 (DM Type 1), Type 2 (DM Type 2) and diabetic subjects suffering from polyneuropathy (DM Type 2 NP+).

As shown in Table 2 and Fig 8, there was no difference (p = 0.13) in the mean (+/-SD) number of dermal CD31 stained vessels per ROI in skin biopsies of healthy volunteers following topical capsaicin at D1 (88.8 +/- 30.0/ mm^2^) and DVar (103.9 +/-33.1/ mm^2^) compared with healthy non-treated controls (117.6+/-72.2/mm^2^) and D54 (132.5+/-64.8/ mm^2^) (Fig 8A). Similar results were observed for the total area of the CD31 staining in the analyzed areas (Fig 8B). Albeit not significant, dermal blood vessels tended to change however in dimensions (length and border) at D1 (0.0006+/-0.0002/mm^2^; 0.002+/-0.001/mm^2^) compared with controls (0.0008+/-0.0005/mm^2^; 0.003+/-0.002/mm^2^). Vessels returned to their normal shape by D54 (0.001+/-0.0005/mm^2^; 0.004+/-0.002/mm^2^; p = 0.21; Fig 8C). The density of CD31 stained vessels in dermis of controls was similar to both that of patients with type 1 and type 2 diabetes who were asymptomatic for neuropathy, and those of patients with diabetes suffering from polyneuropathy (p = 0.96; Control: 118+/-72/mm^2^; DM Type 1: 115+/-44/mm^2^; DM Type 2: 104+/-20/mm^2^; DM Type 2 NP+: 112+/-49/mm^2^) (Fig 8D). No differences were observed in dimensions (Fig 8F) of the vasculature or total CD31-stained area between controls and diabetic patients (Fig 8E, Table 2). These results indicate that capsaicin application induces minor vasoconstriction in dermis, whereas no changes in dermal vasculature were observed in diabetic subjects.

## Discussion

In the present study we aimed at determining the capability of the capsaicin model to truly mimic the morphological manifestations of SFN in diabetes by evaluating NRP-1 receptor expression and vascularization in skin. The anti-human NRP-1 antibody used in this study was shown to be specific as revealed by western blot analysis and immunohistochemistry. A prominent NRP-1 expression was found in dermal vasculature of healthy subjects, most likely arterial as reported by Yuan and colleagues [52] where it contributes to angiogenesis [53]. Besides endothelial NRP-1 expression, non-endothelial immunoreactivity in cells adjacent to vessels was observed, most probably smooth muscle cells, consistent with findings reported by Pellet-Many and coworkers [54]. Cells presenting a fibroblastic phenotype [55] were positive as well. The identification of the different cell types exhibiting NRP-1 expression was however not performed in this study. NRP-1 expression is expected in dermal nerve fibers of the C-type [56], which terminate at the dermal-epidermal junction, although this was not confirmed due to incompatibility of the biopsy processing method applied with PGP9.5 immunostaining. The epidermal NRP-1 receptor expression was restricted to the basal layers in 75% of healthy skin biopsies. Additionally, weak expression of the receptor was identified in supra-basal layers of healthy skin in only 25% of cases. This is in contrast to the expression profile of the NRP-1 receptor reported by Man [57], Riese [58], Shahrabi-Farahani [59] and Kürschat [60] reporting a consistent expression of NRP-1 in all vital epidermal layers. The use of different primary antibodies in these studies or a variable differentiation status of epidermal cells [59] could explain the discrepancy.

By studying vascularization using the universal vessel marker CD31, we report no abnormalities in any of the studied diabetic groups. Although changes to the microvascular network in diabetes are well described [38,40,61,62], this network appeared normal in our limited cohort.

Our results indicate that the topical application of capsaicin negatively influences the presence of NRP-1 in epidermis and dermal non-vascular tissue components. In comparison to the baseline expression of the NRP-1 receptor in healthy volunteers, capsaicin-treated cases showed a significant loss of the receptor in epidermis. In addition, capsaicin induced a decrease of the NRP-1 receptor in dermal non-vascular structures compared to the control expression profile, which could not be confirmed in diabetics. A significant recovery of the dermal NRP-1 receptor was assessed on day 54 after capsaicin was applied. The dermal cell population exhibiting these changes for NRP-1 was however not identified, imposing further investigation. The topical application of capsaicin to the skin initially results in burning pain by C- and A delta-fiber discharges [2], local blood vessel dilation leading to reddening of the skin and edema [63]. Upon repeated application however, sensory nerve fiber degeneration occurs [2,6]. Constriction of smooth muscle cells of the vascular network is conceivable [64] which could explain the vasoconstriction we observed. Cell type-specific differences in capsaicin responsiveness are however described [65]. Evidence of a functional interaction between capsaicin and NRP-1 receptor however does not exist to the best of our knowledge. A chemical NRP-1 epitope break-down and/or subsequent inactivation of the receptor induced by capsaicin cannot be excluded. Whether the epidermal NRP-1 receptor loss attributes to the chemical axotomy is uncertain, and might be unrelated since the epidermal nerve fiber degeneration can be solely attributed to the TRPV1-cytotoxicity induced by capsaicin [11,66].

Interestingly, in contrast to the capsaicin-induced significant loss of epidermal NRP-1 receptor, we found that the epidermal NRP-1 receptor expression pattern changed from solely basal in healthy subjects, to a full epidermal layer expression in 90% of diabetic subjects suffering from polyneuropathy. Our results thus indicate that NRP-1 might have an important role in the development of SFN or inhibits the regeneration of the epidermal nerve fibers [67–70] in diabetes. It is well described that NRP-1 receptor is involved in axon guidance; through binding with Sem3A it induces growth cone collapse and axonal repulsion of several neuronal populations [71,72]. As discussed already by Kou and colleagues [56], Sem3A may restrict C-fiber outgrowth, invasion and thus regeneration in the epidermis. NRP-1 receptor trafficking towards the membranes of all epidermal cells in diabetes as seen in this study and subsequent Sem3A binding, could change the epidermal environment to a nerve fiber repellant bed [48,73]. This would facilitate rapid depolymerization and endocytosis of F-actin, inducing cytoskeletal collapse of the axon [74,75] and thus loss of epidermal nerve fibers in these subjects [6] or simply hinder the regeneration of these fibers into the epidermis [68,70]. The opposite phenomenon has already been described by many scientists in hypersensitivity conditions of the skin, e.g. decreased epidermal expression of Sem3A in atopic dermatitis, psoriasis and itch [56,71,75–77]; all pathologic conditions in which an increased density of epidermal nerve fibers is described. Thorough investigation of Sem3A and NRP-1 expression in skin of a larger cohort of diabetic subjects suffering from neuropathy would reveal their role in the development of peripheral somatosensory deficits in diabetes.

In conclusion, as capsaicin seems to deplete NRP-1 receptor expression in epidermis, it might influence the epidermal microenvironment to become an attractant in favor of rapid nerve fiber regeneration. An axon-repellant epidermal environment seems to be created in diabetic subjects suffering from polyneuropathy sustaining the nerve fiber deficits.

## Abbreviations

BMI: Body mass index
CD31: Cluster of differentiation 31
DX: Day X after capsaicin application
DM: Diabetes mellitus
DNP: Diabetic neuropathy
HbA1C: Glycated hemoglobin level
HEK: Human embryonic kidney
IENFd: Intra-epidermal nerve fiber density
IgG: Immunoglobulin G
kDa: kilo Dalton
LEP: Laser evoked potential
NP: Neuropathy
NRP-1: Neuropilin-1 receptor
NRP-2: Neuropilin-2 receptor
OCT: Optimal cutting tissue compound
PGP9.5: Protein gene product 9.5
QST: Quantitative sensory testing
ROI: Region of interest
SD: Standard deviation
SFN: small fiber neuropathy
TCSS: Toronto Clinical Scoring System
TRPV1: Transient receptor vanilloid 1
Var: variable
VEGF: Vascular endothelial growth factor.
